# Motor Neuron Disease in a Patient With Cervical Spondylotic Myelopathy: Too Much Bad Luck

**DOI:** 10.7759/cureus.12523

**Published:** 2021-01-06

**Authors:** Luis A Robles, Vikram Chakravarthy

**Affiliations:** 1 Neurosurgery, CMQ Premiere Hospital, Puerto Vallarta, MEX; 2 Neurosurgery, Cleveland Clinic Foundation, Cleveland, USA

**Keywords:** amyotrophic lateral sclerosis, cervical spondylotic myelopathy, motor neuron disease

## Abstract

Cervical spondylotic myelopathy (CSM) and amyotrophic lateral sclerosis (ALS) share some clinical findings. Hence, motor neuron disease (MND) should be considered in the differential diagnosis of patients presenting with signs and symptoms of CSM. This unique case demonstrates the coexistence of both conditions in the same patient. The author reports the case of a 74-year-old male who initially underwent posterior cervical decompression and instrumented fusion for cervical myelopathy. He demonstrated postoperative improvement followed subsequently by unexplained neurological deterioration. A repeat MRI showed adequate decompression of the cervical cord and persistence of T2 hyperintense signal in the spinal cord. Based on the presence of signs and symptoms of lower motor neuron disease, electromyography (EMG) was performed demonstrating findings of MND. The presence of MND in a patient with CSM is unique and can be difficult to diagnose based on overlapping symptoms. This case highlights the importance of EMG and the vigilance that spine surgeons need to display to identify ALS or other MND, despite the presence of ongoing cervical myelopathy. In cases where patients show discordant symptoms, further studies should be performed.

## Introduction

Amyotrophic lateral sclerosis (ALS) is a fatal, idiopathic neurodegenerative, and progressive disease of the motor neuron system. It is the most common motor neuron disease (MND) in adults, most frequently seen in patients 50-75 years of age. ALS presents with both upper and lower motor neuron involvement, with severe symptoms of bulbar weakness and eventually respiratory paralysis leading to death occurring three to five years after diagnosis [1].

Cervical spondylotic myelopathy (CSM) is a common degenerative disease caused by spondylosis, leading to spinal cord compression. Typically, it is a progressive condition that is best treated surgically. Patients with CSM usually show a wide variety of upper motor neuron (UMN) signs, including spasticity and hyperreflexia. Both ALS and CSM present with UMN symptoms, especially early in the disease course. It is well known that patients with ALS can initially be misdiagnosed with CSM and vice versa. We present an unusual case of a patient presenting with both MND and CSM.

## Case presentation

**Figure 1:**
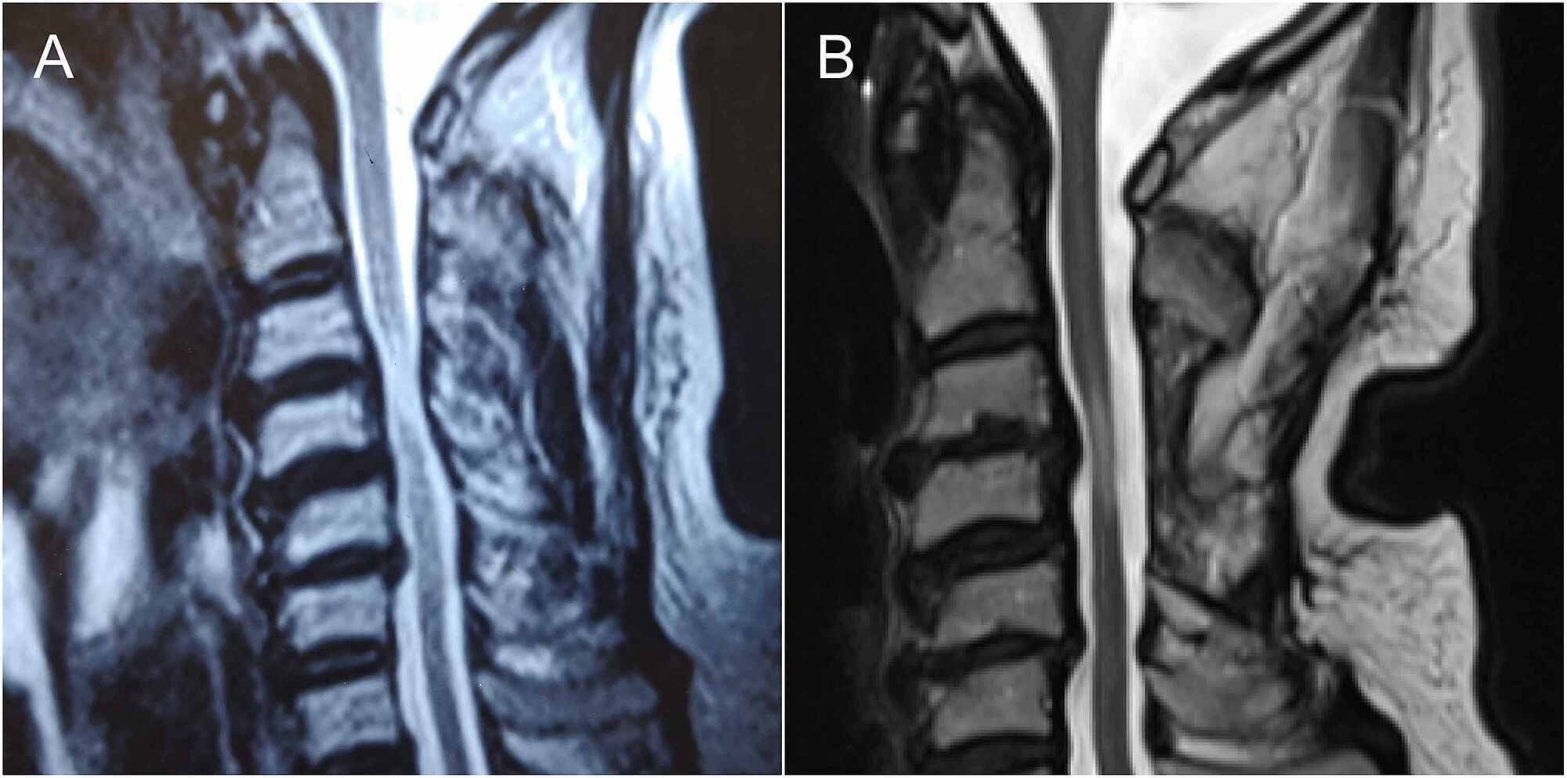
Pre- and postoperative cervical MRI Sagittal MRI T2 weighted. (A) Preoperative image displaying cervical spondylosis characterized by loss of lordosis, disc bulging, and cervical stenosis predominately at C3-4. A hyperintense signal is observed in the spinal cord at C3-4. (B) Postoperative image showing adequate decompression of the cord at C3-4, note the persistence of the hyperintense signal in the same area of the cord.

A 74-year-old male with a four-year history of CSM characterized by quadriparesis, decreased sensation on arm and legs, difficulty passing urine and hyperreflexia. He was treated with posterior cervical decompression and instrumented fusion two years prior at an outside institution. Initial magnetic resonance imaging (MRI) demonstrated cervical stenosis, most significant at C3-4 and a T2 hyperintensity within the spinal cord at that level (Figure 1A).

Postoperatively, he experienced a progressive improvement in the strength of all extremities, regaining the ability to walk. Six months after surgery, his neurological improvement plateaued and subsequently deteriorated. In the following months, the patient continued to decline neurologically, prompting a new cervical MRI. The new MRI demonstrated adequate decompression of the spinal cord and persistence of T2 hyperintense signal in the cervical cord, indicative of spinal cord injury (Figure 1B). Over the next two months, the patient continued to decline in motor function rapidly, with the development of diffuse muscle atrophy in all extremities.

**Figure 2:**
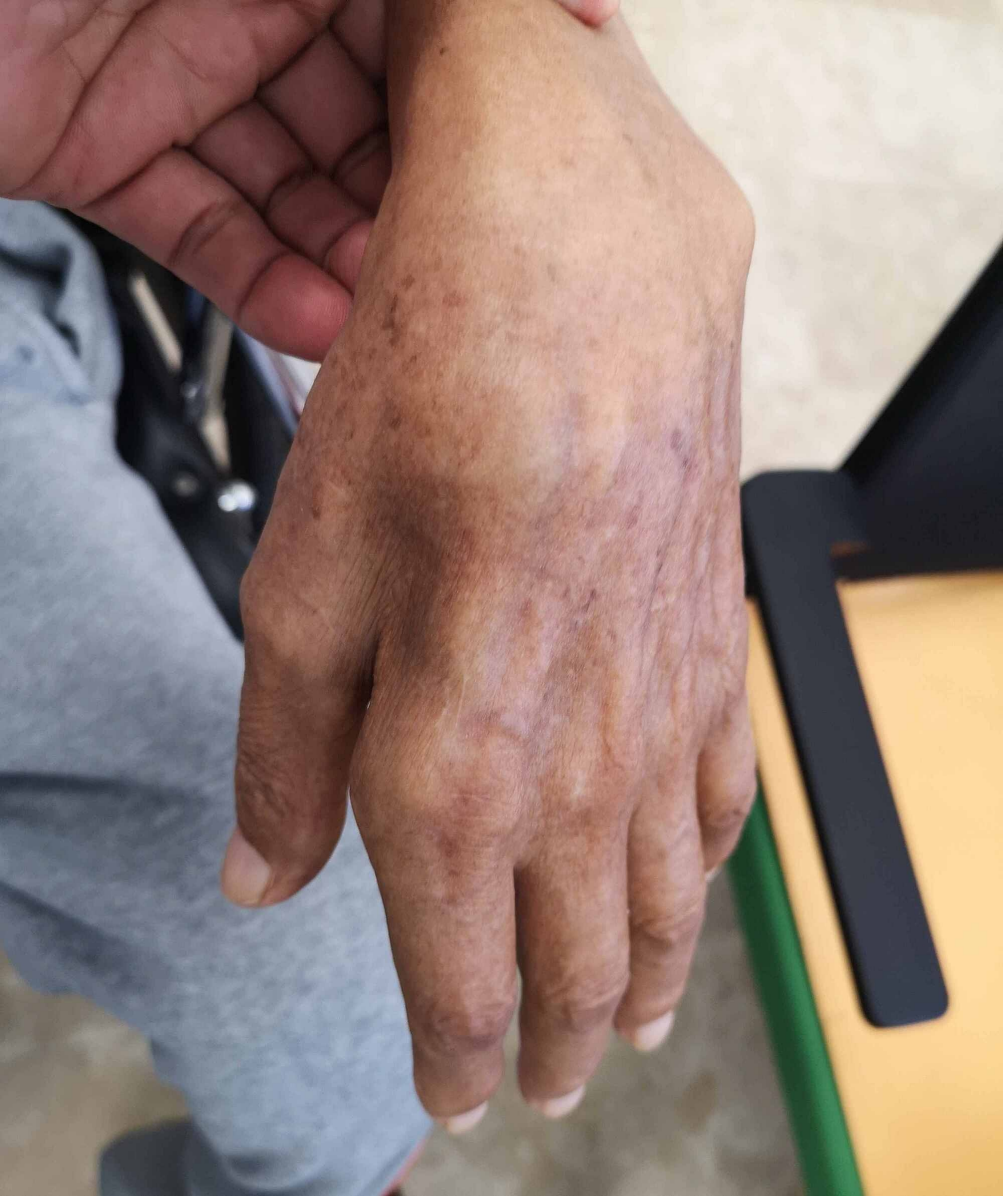
Clinical findings on the patient's hand Image of patient's hand showing severe wasting predominately in the first dorsal interossei muscles.

On examination, the patient was wheelchair-dependent, found to have tongue fasciculations. Upper limbs showed muscle atrophy and bilateral weakness, proximal muscle group strength was three of five, and distal muscle group strength two of five (Medical Research Council {MRC} scale). Severe muscle wasting was observed in both hands, most profoundly in the dorsal interossei muscles (Figure 2).

Lower limbs demonstrated noticeable atrophy and weakness (two of five). Decreased vibratory, positional, and pinprick hypoesthesia was noted in the upper and lower extremities. Areflexia was noted in all extremities.

An electromyography (EMG) and Nerve Conduction Study demonstrated lower motor neuron (LMN) involvement characterized by sharp waves, polyphasic potentials, reduced interferential pattern, and increased firing rate in upper and lower limb muscles. In addition, fasciculation potentials were observed in the genioglossus muscle. Also, The patient was referred to neurology for further evaluation and the diagnosis of anterior horn cell disease was confirmed.

## Discussion

ALS, also known as Lou Gehrig`s disease, is the most common MND that causes progressive weakness, muscle atrophy, leading to death. Most cases of ALS are sporadic, but uncommon genetic cases have been reported in 10% of cases. The clinical hallmark of ALS is the presence of both UMN and LMN findings. The main presentations of ALS include (1) limb-onset ALS with a combination of UMN and LMN signs in the limbs; (2) bulbar-onset ALS, presenting with speech and swallowing difficulties, with extremity symptoms developing later in the disease course; (3) the less common primary lateral sclerosis with pure UMN involvement; and (4) progressive muscular atrophy, with pure LMN involvement [2]. It is reported that many neurological conditions may mimic MND including multifocal motor neuropathy, Kennedy disease, motor neuropathy, cervical myelopathy, and inclusion body myositis to name a few [3].

In the present case, the patient predominantly showed clinical and EMG findings of LMN syndrome, suggestive of progressive muscular atrophy, considered to be a rare presentation of MND [4]. The patient met both the clinical criteria as well as the Awaji EMG criteria for MND [5].

Multiple publications have reported clinical cases where initially CSM was misdiagnosed for ALS and vice versa [6,7]. The coexistence of CSM and ALS as observed in the presented case is rare. MRI findings initially demonstrated a clinical presentation consistent with CSM secondary to spinal cord compression. After surgical decompression, generally, patients improve clinically, until reaching a plateau. However, it is rare for patients to decline neurologically thereafter with a negative compressive pathology on MRI. 

Although the presence of weakness can be caused by either MND or CSM, some findings are specific for one diagnosis compared to the other. In the present case, the patient showed residual symptoms of CSM such as bladder disturbance and sensory symptoms in the extremities. These symptoms, sensory or bowel/bladder deficits usually do not occur in ALS or other MND. On the other hand, he also manifested features of MND such as bulbar symptoms, severe muscle wasting, areflexia, and EMG findings of LMN involvement.

## Conclusions

The presence of CSM and MND in the same patient is a unique situation. This case highlights the importance of including MND in the differential diagnosis of patients with CSM, especially when neurological deterioration occurs after an initial postoperative period of clinical improvement.
